# Synthesis and Cytotoxic Evaluation of Steroidal Copper (Cu (II)) Complexes

**DOI:** 10.1155/2017/4276919

**Published:** 2017-10-18

**Authors:** Yanmin Huang, Erbin Kong, Junyan Zhan, Shuang Chen, Chunfang Gan, Zhiping Liu, Liping Pang, Jianguo Cui

**Affiliations:** ^1^Key Laboratory of Beibu Gulf Environment Change and Resources Utilization, College of Chemistry and Material Science, Guangxi Teachers Education University, Nanning 530001, China; ^2^Guangxi Colleges and University Key Laboratory of Beibu Gulf Oil and Natural Gas Resource Effective Utilization, Qizhou University, Qizhou, China

## Abstract

Using estrone and pregnenolone as starting materials, some steroidal copper complexes were synthesized by the condensation of steroidal ketones with thiosemicarbazide or diazanyl pyridine and then complexation of steroidal thiosemicarbazones or steroidal diazanyl pyridines with Cu (II). The complexes were characterized by IR, NMR, and HRMS. The synthesized compounds were screened for their cytotoxicity against HeLa, Bel-7404, and 293T cell lines in vitro. The results show that all steroidal copper (II) complexes display obvious antiproliferative activity against the tested cancer cells. The IC_50_ values of complexes** 5** and** 12** against Bel-7404 (human liver carcinoma) are 5.0 and 7.0 *μ*M.

## 1. Introduction

Metal-based antitumor drugs play a relevant role in antiblastic chemotherapy [1, 2]. Cisplatin is regarded as one of the most effective drugs [3–8], even if severe toxicities and drug resistance phenomena limit its clinical use [9]. Therefore, in recent years, there has been a rapid expansion in research and development of novel metal-based anticancer drugs in order to improve clinical effectiveness, reduce general toxicity, and broaden the spectrum of activity [10–12].

Copper (Cu) is a transition metal that can exist in oxidised and reduced states. This allows it to participate in redox and catalytic chemistry, making it a suitable cofactor for a diverse range of enzymes and molecules. Cu deficiency or toxicity is implicated in a variety of pathological conditions.

Steroid hormones play an important role in the biochemistry of many cancers; a number of steroidal complexes connected to a metal pharmacophore had been designed and synthesized by many research groups, and their physiological activities were evaluated [13, 14].

Thiosemicarbazones have received considerable attention since the discovery of their cytotoxic activity against cancer cells and bacteriostatic effects [15]. As the disruption of copper homeostasis is a pathological feature of cancer cells, copper complexes had been investigated for their potential applications as anticancer drugs [16]. Cu complexes of thiosemicarbazone (TSC) compounds had been explored as antimalarial, antifungal, antinociceptive, and antibacterial agents [17–19]. Cu complexes of bis(thiosemicarbazones) (CuII(btsc)s) had also been investigated as metallodrugs and diagnostic agents [20]. More recently, Adsule et al. [13] investigated the bioactivity of some new steroidal thiosemicarbazones Cu (II) metal complexes and discovered that some compounds had better antineoplastic activity.

In the present study, some steroidal copper complexes were synthesized by the condensation of steroidal ketones with thiosemicarbazide or diazanyl pyridine and then complexation of steroidal thiosemicarbazones or steroidal diazanyl pyridines with Cu (II). The synthesized compounds were screened for their cytotoxicity against HeLa, Bel-7404, and 293T cell lines in vitro.

## 2. Materials and Methods

### 2.1. Materials

The sterols were purchased from Sinopharm Chemical Reagent Co., Ltd., Shanghai, China. All chemicals and solvents are of analytical grade from commercial sources. All solvents were used without further purification unless otherwise specified.

### 2.2. Instrumentation and Methods

Melting points were determined on an X4 apparatus (Beijing Tech Instrument Co. Ltd., Beijing, China) and were uncorrected. The ^1^H and ^13^C NMR spectra were recorded in CDCl_3_ on a Bruker AV-600 spectrometer at working frequencies of 600 and 150 MHz and a Bruker AV-300 spectrometer at working frequencies of 300 and 75 MHz, respectively. Chemical shifts are expressed in parts per million (*δ*) values and coupling constants (*J*) in Hertz. Infrared spectra were measured with a Thermo Scientific Nicolet IS-10 Spectrophotometer (Thermo Scientific, USA). HREIMS was measured on an Agilent 6210 TOFMS instrument (Agilent Technologies, USA). The cell proliferation assay was undertaken by a MTT method using 96-well plates on a MLLTISKAN MK3 analysis spectrometer (Thermo Scientific, Shanghai, China).

Compounds** 1** (L^1^) and** 2** (L^2^) were prepared according to the method of [21].

### 2.3. Synthesis

#### 2.3.1. General Procedure for Preparation of Steroidal Thiosemicarbazones

Steroidal ketone (0.38 mmol) was dissolved in 40 mL 95% ethanol. After the solution was heated to 65°C, a few drops of glacial acetic acid were added to adjust pH to 3–5, and thiosemicarbazide (1.70 mmol) was added. The mixture was stirred at 60–70°C for 6 h (the progress of the reaction was monitored by TLC, *V*_ethyl  acetate_ : *V*_petroleum  ether_ = 1 : 2). Then, the reaction was terminated and majority of solvent was evaporated under reduced pressure. Suitable amount of water was added to the reaction mixture, and the product was extracted with CH_2_Cl_2_. The combined extract was washed with saturated NaHCO_3_ solution, water, and saturated brine, dried with anhydrous sodium sulfate, and evaporated under reduced pressure. The resulting residue was separated by the column chromatography using a mixture of ethyl acetate : petroleum ether (1 : 2) to give target products.

*3β-Hydroxypregnenolone-20-semicarbazone (* ***3***, *L*^3^). White solid, Yield: 78.0%; m.p. 239–241°C; IR (KBr) *ν*/cm^−1^: 3427, 3362, 3219, 3162, 2914, 2888, 1605, 1515, 1433, 1282, 1254; ^1^H NMR (CDCl_3_, 300 MHz)*δ*: 0.60 (3H, s, 18-CH_3_), 0.65 (3H, d, *J* = 6.6, 21-CH_3_), 1.21 (3H, s, 19-CH_3_), 3.56–3.45 (1H, m, C_3_-H), 5.37 (1H, brs, C_6_-H), 6.238 (1H, s, -NH_2_), 7.22 (1H, s, -NH_2_), 8.51 (1H, s, -NH-); ^13^C NMR (CDCl_3_, 75 MHz)*δ*: 179.0 (-C=S), 153.2 (-C=N), 140.8 (5-C), 121.4 (6-C), 71.1 (3-C), 59.0 (14-C), 56.5 (17-C), 56.4 (9-C), 50.1 (13-C), 42.9 (4-C), 48.8 (12-C), 42.2 (8-C), 37.2 (1-C), 36.5 (10-C), 31.8 (7-C), 31.6 (16-C, 2-C), 24.1 (15-C), 21.0 (11-C), 19.4 (21-C), 17.3 (19-C), 13.2 (18-C); HREIMS:* m*/*z* 390.2577 [M+H]^+^ (calcd for C_22_H_36_N_3_OS, 390.2579). 

*3β-Acetyloxypregnenolone-20-semicarbazone (* ***4***, *L*^4^). White solid, Yield: 74.5%; m.p. 254-255°C; IR (KBr)*ν*/cm^−1^: 3414, 1726, 1589, 1506, 1437, 1369, 1252, 846; ^1^H NMR (300 MHz, CDCl_3_)*δ*: 0.58 (s, 3H, 18-CH_3_), 1.02 (s, 3H, 19-CH_3_), 1.91 (s, 3H, 21-CH_3_), 2.03 (s, 3H, COCH_3_), 4.65–4.55 (m, 1H, C3-H), 5.38 (d, 1H, *J* = 4.5, C5-H), 6.52 (br s, 1H, -NH_2_), 7.22 (br s, 1H, -NH_2_), 8.63 (s, 1H, -NH); ^13^C NMR (75 MHz, CDCl_3_)*δ*: 13.3 (19-C), 17.5 (18-C), 19.3 (11-C), 21.0 (CH_3_CO), 21.4 (21-C), 23.3 (15-C), 24.1 (16-C), 27.7 (2-C), 31.7 (8-C), 32.0 (7-C), 36.6 (10-C), 37.0 (1-C), 38.1 (4-C), 38.8 (12-C), 44.2 (13-C), 50.0 (9-C), 56.4 (17-C), 59.0 (14-C), 73.8 (3-C), 122.3 (6-C), 139.7 (5-C), 153.5 (20-C), 170.6 (C=O), 178.9 (C=S); HREIMS:* m/z* 432.2633 [M+H]^+^ (calcd for C_24_H_38_N_3_O_2_S, 432.2685).

#### 2.3.2. General Procedure for Preparation of Steroidal Diazanyl Pyridine

A mixture of steroidal ketone (1 mmol) and diazanyl pyridine (1 mmol) in 95% ethanol (30 mL) was stirred at 70–80°C for 6 h. After completion of the reaction, the majority of solvent was evaporated and some water was added to this solution. The mixture was extracted with CH_3_COOC_2_H_5_ and the extract was washed with saturated brine, dried with anhydrous sodium sulfate, and evaporated under reduced pressure. The resulting residue was chromatographed on a column of silica gel with mixture of petroleum ether/ethyl acetate (1 : 1) to give steroidal diazanyl pyridine. 

*3β-Hydroxyoestrone-17-(2*′*-diazanyl)pyridine ( ****9***, *L*^9^). Yellow solid, Yield: 56.0%; m.p. 269–271°C; IR (KBr)*ν*/cm^−1^: 3361, 2935, 1601, 1576, 1444, 995, 871, 768; ^1^H NMR (600 MHz, DMSO)*δ*: 0.85 (3H, s, 18-CH_3_), 2.36–2.30 (2H, m, C6-H and C9-H), 2.54 (1H, dd, *J* = 18.6, 9.0, C6-H), 2.70-2.69 (2H, m, C16-H), 6.45 (1H, d, *J* = 2.4, C4-H), 6.52 (1H, dd, *J* = 8.4, 2.4, C2-H), 6.67 (1H, t, *J* = 6.0, 5′-Py-H), 7.06 (1H, d, *J* = 8.4, C1-H), 7.07 (1H, d, *J* = 8.4, 3′-Py-H), 7.56 (1H, td, *J* = 8.4, 1.8, 4′-Py-H), 8.04 (1H, d, *J* = 3.6, 6′-Py-H), 8.98 (1H, s, -NH), 9.04 (1H, s, -OH); ^13^C NMR (150 MHz, DMSO)*δ*: 17.3 (18-C), 23.0 (11-C), 26.1 (15-C), 26.9 (16-C), 29.2 (7-C), 34.4 (6-C), 38.0 (12-C), 40.1 (8-C), 43.8 (9-C), 44.2 (13-C), 52.2 (14-C), 106.4 (3′-Py-C), 112.8 (2-C), 114.3 (4-C), 115.0 (5′-Py-C), 126.1 (1-C), 130.3 (10-C), 137.2 (4′-Py-C), 137.6 (5-C), 147.5 (6′-Py-C), 155.0 (3-C), 158.3 (2′-Py-C), 162.9 (17-C); HREIMS: [M+H]^+^ 362.2250 (calcd for C_23_H_28_N_3_O, 362.2232). 

*3β-Hydroxypregnenolone-20-(2*′*-diazanyl)pyridine ( ****10***, *L*^10^). Yellow solid, Yield: 78.8%; m.p. 234-235°C; IR(KBr) *ν*/cm^−1^: 3406, 2932, 1599, 1574, 1442, 838, 768; ^1^H NMR (600 MHz, DMSO)*δ*: 0.55 (3H, s, 18-CH_3_), 0.94 (3H, s, 19-CH_3_), 1.89 (3H, s, 20-CH_3_), 3.35–3.20 (1H, m, C3-*α*H), 4.64 (1H, br s, NH), 5.27 (1H, s, C6-H), 6.69 (1H, t, *J* = 6.6, 5′-pyridine-H), 7.06 (1H, d, *J* = 7.2, 3′-pyridine-H), 7.57 (1H, t, *J* = 7.2, 4′-pyridine-H), 8.05 (1H, d, *J* = 6.6, 6′-pyridine-H), 9.07 (s, 1H, -OH); HREIMS:* m*/*z* 408.3024 [M+H]^+^ (calcd for C_26_H_38_N_3_O, 408.3015).

#### 2.3.3. General Procedure for Preparation of Copper Complexes

Steroidal ligand (0.1 mmol) and 0.1 mmol CuCl_2_·2H_2_O were added to 8 mL of methanol. The mixture was stirred for 5 hour at 70°C. The reaction was terminated when large precipitant emerged. The resulting suspension was filtered, washed with ethyl acetate and water, and dried in a desiccator over phosphorus pentoxide to give target products. 

*[CuL* ^1^ *Cl*_2_ *] (Compound* ***5***). Compound** 5** is a mixture of (*S*)- and (*R*)-configuration isomer (**5**-S : **5**-R = 1.7 : 1, ^1^H NMR data). Gray yellow solid, Yield: 55%; m.p. 245–247°C; IR (KBr)*ν*/cm^−1^: 3441, 1604, 1541, 1409, 1452, 1280, 811, 616; ^1^H NMR (300 MHz, DMSO)*δ*: 0.81 (s, 1.07H, 18-CH_3_,* R-*), 0.86 (s, 1.71H, 18-CH_3_,* S-*), 6.44 (s, 1H, C4-H), 6.50 (d, 1H, *J* = 4.5, C2-H), 7.05 (d, 1H, *J* = 4.5, C1-H), 7.77 (s, 0.19H, -NH_2_,* R-*), 8.00 (s, 0.31H, -NH_2_,* S-*), 8.65 (s, 0.36H, -NH_2_,* S-*), 8.85 (s, 0.25H, -NH_2_,* R-*), 9.03 (s, 1H, -OH), 10.30 (s, 0.34H, -NH-,* S-*), 10.64 (s, 0.20H, -NH-,* R-*).

*[CuL* ^2^ *Cl*_2_ *] (Compound* ***6***). Compound** 6** is a mixture of (*S*)- and (*R*)-configuration isomer (**6**-S : **6**-R = 1.5 : 1, ^1^H NMR data). Gray solid, Yield: 66.7%; m.p. 189-190°C; IR (KBr) *ν*/cm^−1^: 3416, 2927, 1604, 1534, 1496, 1447, 876, 816, 751; ^1^H NMR (600 MHz, DMSO): 0.85 (s, 1.85H, 18-CH_3_,* S-*), 1.01 (s, 1.25H, 18-CH_3_,* R-*), 2.21 (s, 3H, COCH_3_), 6.79 (s, 1H, C4-H), 6.83 (d, 1H, *J* = 6.0, C2-H), 7.29 (s, 1H, *J* = 6.0, C1-H), 7.73 (s, 0.38H, -NH_2_), 8.04 (s, 0.45H, -NH_2_,* S-*), 8.68 (s, 0.48H, -NH_2_,* S-*), 8.84 (s, 0.33H, -NH_2_,* R-*), 9.023 (s, 1H, -OH), 10.31 (s, 0.40H, -NH-,* S-*), 10.63 (s, 0.33H, -NH-,* R-*); ^13^C NMR (150 MHz, DMSO)*δ*: 16.5 (18-C), 20.6 (CH_3_CO), 22.3 (11-C), 26.1 (15-C), 26.4 (16-C), 28.6 (7-C), 28.8 (6-C), 33.5 (12-C), 37.1 (8-C), 37.5 (8-C), 43.1 (9-C), 44.6 (13-C), 51.6 (14-C), 118.6 (2-C), 121.2 (4-C), 126.0 (1-C), 136.8 (5-C), 137.4 (10-C), 148.0 (3-C), 154.7 (17-C), 169.1 (COCH_3_), 170.1 (C=S), 171.3 (C=S). 

*[CuL* ^3^ *Cl*_2_ *] (Compound* ***7***). Compound** 7** is a mixture of (*S*)- and (*R*)-configuration isomer (**7**-S : **7**-R = 1 : 1.5, ^1^H NMR data). Gray solid, Yield: 60.2%; m.p. 189–191°C; IR (KBr)*ν*/cm^−1^: 3319, 1604, 1531, 1434, 1369, 1290, 1050, 950; ^1^H NMR (600 MHz, DMSO)*δ*: 0.51 (s, 1.2H, 18-CH_3_,* S-*), 0.68 (s, 1.8H, 18-CH_3_,* R-*), 0.91 (s, 3H, 19-CH_3_), 1.96 (s, 1.2H, 21-CH_3_,* S-*), 2.00 (s, 1.8H, 21-CH_3_,* R-*), 3.23–3.12 (m, 1H, C3-H), 4.61 (s, 1H, OH), 5.24 (s, 1H, C6-H), 7.75 (s, 0.6 H, -NH_2_,* R-*), 8.01 (s, 0.4H, -NH_2_,* S-*), 8.73 (s, 0.4H, -NH_2_,* S-*), 8.95 (s, 0.6H, -NH_2_,* R-*), 10.29 (s, 0.4H, -NH-,* S-*), 10.89 (s, 0.6H, -NH-,* R-*); ^13^C NMR (150 MHz, DMSO)*δ*: 12.7 (19-C), 18.7 (18-C), 20.3 (11-C), 20.8 (21-C), 21.9 (15-C), 23.7 (16-C), 27.0 (2-C), 31.0 (8-C), 31.1 (7-C), 35.8 (1-C), 36.6 (10-C), 37.4 (4-C), 41.9 (12-C), 43.0 (13-C), 49.2 (9-C), 55.7 (17-C), 58.2 (14-C), 69.7 (3-C), 121.7 (6-C), 139.2 (5-C), 140.9 (20-C), 169.5 (C=S); HREIMS:* m/z* 521.1197 [M−H]^−^ (calcd for C_22_H_34_Cl_2_CuN_3_OS, 521.1196). 

*[CuL* ^4^ *Cl*_2_ *] (Compound* ***8***). Compound** 8** is a mixture of (*S*)- and (*R*)-configuration isomer (**8**-S : **8**-R = 1.3 : 1, ^1^H NMR data). Gray solid, Yield: 75%; m.p. 163–165°C; IR (KBr)*ν*/cm^−1^: 3424, 1723, 1596, 1534, 1432, 1364, 1040; ^1^H NMR (600 MHz, DMSO)*δ*: 0.50 (s, 1.7H, 18-CH_3_,* S-*), 0.671 (s, 1.3H, 18-CH_3_,* R-*), 0.94 (s, 3H, 19-CH_3_), 1.95 (s, 1.7H, 21-CH_3_,* S-*), 1.98 (s, 1.3H, 21-CH_3_,* R-*), 2.24 (s, 3H, COCH_3_), 7.754 (s, 0.43H, -NH_2_,* R-*), 7.962 (s, 0.49H, -NH_2_,* R-*), 8.666 (s, 0.51H, -NH_2_,* S-*), 8.929 (s, 0.52H, -NH_2_,* S-*), 10.276 (s, 0.56H, -NH,* S-*), 10.881 (s, 0.40H, -NH,* R-*); ^13^C NMR (150 MHz, DMSO)*δ*: 12.7 (19-C), 18.7 (18-C), 20.3 (11-C), 20.8 (21-C), 21.9 (15-C), 23.7 (16-C), 27.0 (2-C), 31.0 (8-C), 31.1 (7-C), 35.8 (1-C), 36.6 (10-C), 37.4 (4-C), 41.9 (12-C), 43.0 (13-C), 49.2 (9-C), 55.7 (17-C), 58.2 (14-C), 69.7 (3-C), 121.7 (6-C), 139.2 (5-C), 140.9 (20-C), 169.5 (C=S). 

*[CuL* ^9^ *Cl*_2_ *] (Compound* ***11***). Gray solid, Yield: 62.3%; m.p. 210–212°C; IR (KBr)*ν*/cm^−1^: 3441, 3324, 2962, 1654, 1611, 1559, 1534, 1501, 1442, 916, 848, 744; ^1^H NMR (600 MHz, DMSO): 1.51 (3H, s, 18-CH_3_), 2.84–2.73 (3H, m, C16-H and C6-H), 6.49 (1H, s, C4-H), 6.56 (1H, br s, C2-H), 7.12 (1H, d, *J* = 6.0, 5′-Py-H), 7.45 (1H, br s, 3′-Py-H), 7.86 (1H, br s, 4′-Py-H), 8.08 (1H, d, *J* = 8.4, C1-H), 8.74 (1H, br s, 6′-Py-H), 9.11 (1H, s, -NH); ^13^C NMR (150 MHz, DMSO)*δ*: 16.1 (18-C), 20.6 (15-C), 20.8 (11-C), 25.9 (7-C), 26.1 (6-C), 29.3 (12-C), 35.9 (16-C), 38.3 (8-C), 42.4 (13-C), 65.0 (14-C), 113.1 (2-C), 114.7 (4-C), 115.3 (3′-Py-C), 117.2 (5′-Py-C), 125.2 (4′-Py-C), 126.2 (1-C), 129.3 (10-C), 133.5 (6′-Py-C), 137.0 (5-C), 143.9 (2′-Py-C), 146.2 (17-C), 155.3 (3-C). 

*[CuL* ^10^ *Cl*_2_ *] (Compound* ***12***). Green solid, Yield: 30.7%; m.p. 224–225°C; ^1^H NMR (600 MHz, DMSO)*δ*: 0.96 (3H, s, 18-CH_3_), 1.00 (3H, s, 19-CH_3_), 2.08 (3H, s, 20-CH_3_), 3.37–3.18 (1H, m, C3-*α*H), 4.63 (1H, s, OH), 5.24 (1H, s, C6-H), 6.21 (0.36H, t, *J* = 4.8), 6.33 (0.33H, d, *J* = 6.6, 5′-Py-H), 7.32 (0.33H, br s, 4′-Py-H), 7.69 (0.32H, dd, *J* = 24.6, 6.6, 3′-Py-H), 8.49 (0.60H, s, 6′-Py-H), 9.07 (0.60H, s), 9.23 (0.60H, d, *J* = 6.6), 9.79 (0.57H, s, -NH); ^13^C NMR (150 MHz, DMSO) *δ*: 14.2 (21-C), 18.9 (18-C), 19.7 (11-C), 19.8 (19-C), 20.3 (15-C), 22.2 (16-C), 30.7 (8-C), 31.0 (2-C), 31.1 (7-C), 35.8 (10-C), 36.6 (1-C), 37.8 (12-C), 41.9 (13-C), 42.8 (4-C), 49.2 (17-C), 55.4 (9-C), 57.8 (14-C), 69.7 (3-C), 117.8 (3′-Py-C), 119.8 (5′-Py-C), 122.0 (6-C), 129.2 (4′-Py-C), 140.9 (5-C), 141.0 (6′-Py-C), 149.9 (20-C), 151.9 (2′-Py-C).

### 2.4. Cytotoxicity Assay

The antiproliferative activity of all Cu(II) metal complexes and steroidal thiosemicarbazones on Bel-7404 (human liver carcinoma), HeLa (human cervical carcinoma), and HEK-293T (normal kidney epithelial) cell lines was determined by using the MTT method and cisplatin as a positive control. The detailed procedure had been reported in our previous work [22].

## 3. Results and Discussion

### 3.1. Synthesis and Characterization

**Scheme 1:**
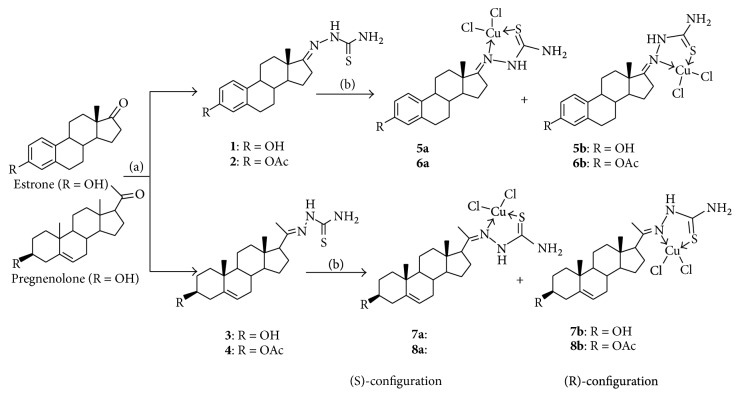
Synthesis of complexes** 5**–**8**. Reagents and conditions: (a) thiosemicarbazide, acetic acid, and ethanol; (b) CuCl_2_·2H_2_O, CH_3_OH/CHCl_3_ = 1 : 1.

The synthetic route and the structures of complexes** 5**–**8** are outlined in Scheme 1. The steroidal thiosemicarbazones** 1**–**4** were obtained by reacting estrone and pregnenolone or their ester with thiosemicarbazide and then the reaction of compounds** 1**–**4** with CuCl_2_·2H_2_O gave steroidal copper (Cu (II)) complexes** 5**–**8** as a mixture of (*R*)- and (*S*)-configuration isomers, respectively. The structures of** 5**–**8** are confirmed by analysis of IR, NMR, and HRMS. Compared with the signal of -NH- in ^1^H NMR for ligand** 3**, the proton chemical shift of -NH- for compound** 7** migrates to*δ* 10.29 (s, 0.4H) and 10.89 (s, 0.6H) ppm of downfield from*δ* 8.51 ppm of upfield due to the effect of Cu (II) and demonstrates the formation of L^3^-Cu (II) complex. The resonances showing of 10.29 and 10.89 ppm belongs to the chemical shift of (*S*)- and (*R*)-**7,** respectively, and illustrates further that compound** 7** is the mixture of (*S*)- and (*R*)-configuration isomers (**7**-S : **7**-R = 1 : 1.5, ^1^H NMR data) from the chemical shift of 18-CH_3_ and 21-CH_3_ (18-CH_3_: 0.51 (1.2H,* S-*), 0.68 (1.8H,* R-*) ppm; 21-CH_3_: 1.96 (1.2H,* S-*), 2.00 (1.8H,* R-*) ppm).

**Scheme 2:**
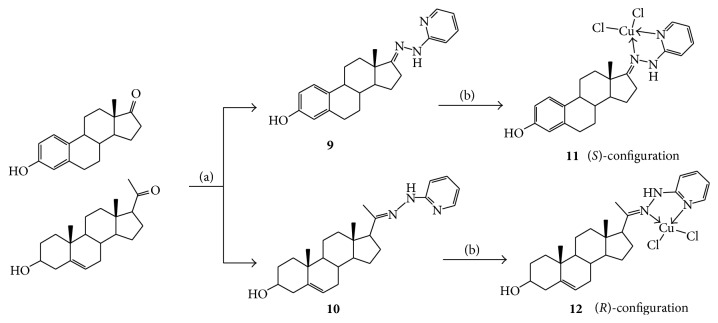
Synthesis of complexes** 11**-**12**. Reagents and conditions: (a) 2-hydrazinopyridine, acetic acid, and ethanol; (b) CuCl_2_·2H_2_O, CH_3_OH/CHCl_3_ = 1 : 1.

In order to investigate the effect of different ligand on the antiproliferative activity of complexes, 3*β*-hydroxyoestrone-17-(2′-diazanyl)pyridine-Copper(II)** 11** and 3*β*-Hydroxypregnenolone-20-(2′-diazanyl) pyridine-Copper (II)** 12** were synthesized according to Scheme 2. Ligands** 9** and** 10** were obtained as a (*E*)-configuration by reacting estrone or pregnenolone with 2-hydrazinopyridine. Furthermore, the reaction of compounds** 9** and** 10** with CuCl_2_·2H_2_O gave steroidal copper (Cu (II)) complexes** 11** and** 12** as (*S*)- and (*R*)-configuration, respectively. The structures of** 11** and** 12** were confirmed by analysis of IR, NMR, and HRMS.

### 3.2. Cytotoxic Activity In Vitro

The antiproliferative activities of all steroidal Cu(II) metal complexes were determined in vitro on Bel-7404 (human liver carcinoma), HeLa (human cervical carcinoma), and 293T (normal kidney epithelial) cell lines. The MTT method was used to assay the antiproliferative activity and cisplatin was used as a positive control. The results are summarized as IC_50_ values in *μ*M in Table 1.

From the data shown in Table 1, all steroidal copper (Cu (II)) complexes show an obvious antiproliferative activity against the tested cancer cells. The compounds** 5**–**7** display a better activity to Bell-7404 and HeLa cells compared to that of cisplatin. Comparing the antiproliferative activity of steroidal thiosemicarbazone ligands with that of their copper (II) complexes, we can see that steroidal thiosemicarbazone copper (II) complexes show a better inhibiting activity compared to their homologous ligands (**1** versus** 5** and** 2** versus** 6**). Particularly, complexes** 5** and** 7** show an excellent antiproliferative activity against Bel-7404 cells with the IC_50_ values of 5.0 and 9.5 *μ*M, and complexes** 6** and** 7** possess IC_50_ values of 7.7 and 6.8 *μ*M against HeLa cells.

Comparing compound** 7** with compound** 8**, we can observe that after 3-hydroxyl group of** 7** was converted into 3-acetoxy group (compound** 8**), the antiproliferative activity of the compound was remarkably decreased and the cytotoxicity to normal cells 293T was increased. The result shows that 3-hydroxyl of the compound to the antiproliferative activity plays an important role.

Unfortunately, these steroidal copper (Cu (II)) complexes to normal kidney epithelial cells (293T) show similar cytotoxicity except for compound** 5** which exhibits a smaller activity to 293T cells compared to cisplatin (27 *μ*M versus 10.3 *μ*M).

## 4. Conclusion

In conclusion, using estrone and pregnenolone as starting materials, through different chemical methods, some steroidal copper (II) complexes were synthesized and characterized by IR, NMR, and HRMS. Their antiproliferative activities were assayed by MTT method. The results show that all steroidal copper (II) complexes display obvious antiproliferative activity against the tested cancer cells, and compounds** 5**–**7** show better cytotoxicity compared to a positive control, cisplatin. Among them, complexes** 5** and** 12** show an excellent antiproliferative activity against Bel-7404 cells with the IC_50_ values of 5.0 and 7.0 *μ*M, and complexes** 6** and** 7** possess IC_50_ values of 7.7 and 6.8 *μ*M against HeLa cells. The result may be useful for the design of novel chemotherapeutic drugs.

## Figures and Tables

**Table 1:** Cytotoxicity^a^ of steroidal thiosemicarbazone and its Cu-complexes in vitro (IC_50_: *µ*M)^b^.

Compounds	Bel-7404	HeLa	293T
1	19	34	ND
2	>200	42	>200
5	5.0	11	27
6	13	7.7	11
7	9.5	6.8	15.3
8	14.1	10.6	9.1
11	14	ND	14
12	7.0	ND	9.0
Cisplatin	23.2	10.1	10.3

^a^Cytotoxicity as IC_50_ for each cell line is the concentration of compound which reduced by 50% the optical density of treated cells with respect to untreated cells using the MTT assay. ^b^Data represent the mean values of the three independent determinations. ND: not determined.
